# Erk2 but not Erk1 regulates crosstalk between Met and EGFR in squamous cell carcinoma cell lines

**DOI:** 10.1186/s12943-015-0319-z

**Published:** 2015-03-04

**Authors:** Simone Gusenbauer, Emanuele Zanucco, Pjotr Knyazev, Axel Ullrich

**Affiliations:** Department of Molecular Biology, Max Planck Institute of Biochemistry, Am Klopferspitz 18, D-82152, Martinsried, Germany

**Keywords:** Erk2, Met, EGFR, Receptor-crosstalk

## Abstract

**Background:**

Squamous cell carcinoma (SCC) is the most common type of tongue and larynx cancer and a common type of lung cancer. In this study, we attempted to specifically evaluate the signaling pathway underlying HGF/Met induced EGFR ligand release in SSCs. The Met proto-oncogene encodes for a tyrosine kinase receptor which is often hyperactivated in human cancers. Met activation correlates with poor patient outcome. Several studies revealed a role of Met in receptor-crosstalk inducing either activation of other receptors, or inducing their resistance to targeted cancer treatments. In an epithelial tumor cell line screen we recently showed that the Met ligand HGF blocks the EGFR tyrosine kinase and at the same time activates transcriptional upregulation and accumulation in the supernatant of the EGFR ligand amphiregulin (Oncogene 32:3846–56, 2013). In the present work we describe the pathway responsible for the amphiregulin induction.

**Findings:**

Amphiregulin is transcriptionally upregulated and is released into the supernatant. We show that Erk2 but not Erk1 mediates amphiregulin upregulation upon treatment with monocyte derived HGF. A siRNA knockdown of Erk2 completely abolishes amphiregulin release in squamous cell carcinomas.

**Conclusions:**

These results identify Erk2 as the key downstream signal transducer between Met activation and EGFR ligand upregulation in squamous cell carcinoma cell lines derived from tongue, larynx and lung.

**Electronic supplementary material:**

The online version of this article (doi:10.1186/s12943-015-0319-z) contains supplementary material, which is available to authorized users.

## Findings

A common feature of many human epithelial cancers is a constitutive activation of the Ras/Raf/MEK/Erk signal transduction pathway [1-4]. This hyperactivation can be either induced via activating mutations in members of this pathway or via binding of extracellular signaling molecules to a receptor on the cell-surface. These extracellular signaling molecules include cytokines, extracellular matrix components, GPCR ligands, neurotransmitters and growth factors. Growth factors bind to specific cell-surface receptors harboring intrinsic protein kinase activity, the so called receptor tyrosine kinases (=TK). Following ligand binding, growth-factor receptor tyrosine kinases such as Met, the EGF receptor family, the fibroblast growth factor receptor family or the vascular endothelial growth factor receptor family become activated and recruit intracellular adaptor proteins to the cytoplasmic tails of the activated receptors. A signaling cascade is triggered involving Ras, Raf and MEK. Activated MEK activates the extracellular signal regulated kinases 1 and 2 (Erk1/2) by phosphorylation of Threonine and Tyrosine residues within their activation loop. Once Erk1 and Erk2 are phosphorylated they regulate the transcription of genes involved in a range of fundamental cellular processes including cell survival, proliferation, motility, and differentiation [5]. The two isoforms Erk1 and Erk2 share a high level of amino acid identity (88%), are ubiquitously expressed in mammalian cells, are coactivated in response to extracellular stimuli [6,7] and recognize the same substrates [8-11]. However various studies describe a nonredundant function of Erk1 and Erk2. Analyses of mouse models show that disruption of the Erk2 gene leads to early embryonic lethality, while disruption of Erk1 does not [12,13]. *In vitro* studies have also suggested that Erk1 and Erk2 may exert distinct functions in certain cellular contexts. For example, a knockdown of Erk2 expression restrains hepatocyte cell division, whereas Erk1 silencing specifically improves long-term hepatocyte survival [14,15]. In breast epithelial cells Erk2 but not Erk1 induces epithelial-to-mesenchymal transformation [16]. Other studies reported that osteosarcoma cells regulate the expression of gp130 via Erk2 [17]. Furthermore it has been reported that siRNA knockdown of Erk1 in fibroblasts enhances Erk2 signaling and results in enhanced cell proliferation [18].

In our study we reveal an Erk2 dependent crosstalk between tumor stroma associated HGF/Met signaling and tumor cell associated EGFR signaling. HGF is a frequently detected ligand in the tumor stroma, mainly released by tumor-associated macrophages (TAMs) and by stromal fibroblasts [19-21]. Met receptor activation in cancer cells upon HGF binding, was shown to trigger several pro-tumorigenic pathways [22-25]. However, the complex crosstalk between epithelial tumor cells and stromal cells is yet poorly understood. Several studies have shown diverse mechanisms of transactivation between Met and the EGF receptor family [26-34]: the hyperactivation of Met, for example, was shown to play a role in resistance formation to EGF-receptor-family-blocking agents [26,27,32]. Scheving et al. demonstrated that inhibition of EGFR TK blocks HGF-induced DNA synthesis in primary hepatocytes, indicating that the proliferative actions of HGF may be secondary via new synthesis or processing of EGFR ligands [29]. Similarly, Spix et al. blocked HGF-induced scattering of human corneal limbal epithelial cells with an EGFR TK inhibitor [30]. Finally Reznik and coworkers demonstrated that HGF stimulation of glioblastoma cells induces EGFR activation via new transcription of EGFR ligands [31].

**Figure 1:**
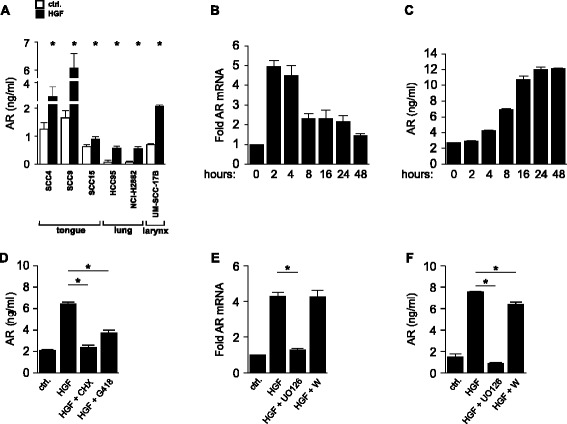
**The MAPK pathway regulates amphiregulin induction and amphiregulin release upon HGF stimulation depends on amphiregulin protein synthesis. (A)** Quantification of amphiregulin protein release in different SCC cell lines treated with HGF for 24 h. Ligand release was assayed using sandwich ELISA. Error bars indicate SEM of three independent experiments. **(B)** Quantification of amphiregulin mRNA induction. SCC9 cells were treated with 100 ng/ml HGF for the indicated time points. Data represent the increase of amphiregulin normalized to HPRT1 cDNA. Values are shown as mean ± SD (n = 2). **(C)** Quantification of amphiregulin protein release. Values are shown as mean ± SD (n = 2). **(D)** Quantification of amphiregulin protein release. SCC9 cells were treated with 100 ng/ml HGF and with the translation inhibitors cycloheximide (=CHX; 1 μg/ml) and geneticin (=G418; 1 mg/ml) for 24 h. Ligand release into the supernatant was assayed using sandwich ELISA. Error bars indicate SEM of three independent experiments. **(E)** Quantification of amphiregulin mRNA induction. SCC9 cells were pretreated with 5 μM of the MEK inhibitor UO126 and 50 nM of the PI3K inhibitor wortmannin (=W) for 15 min before 2 h HGF treatment. **(F)** Quantification of amphiregulin protein release. SCC9 cells were pretreated for 15 min with UO126 and wortmannin before 24 h HGF treatment. Error bars indicate SEM of three independent experiments. Asterisks indicate statistically significant repression or induction (*p* < 0.05, paired *t*-test).

Here, we attempted to specifically investigate the signaling pathway underlying HGF/Met induced EGFR ligand release in SCCs derived from different tissues. Amphiregulin protein release upon HGF stimulation could be observed in SCCs of the tongue, lung and larynx (Figure 1A). In order to investigate which signal transducer downstream of Met activation mediates the upregulation of amphiregulin, we used, due to the high amphiregulin production, SCC9 cells as a preliminary model system. The amphiregulin transcript induction peaked within the first two hours after HGF stimulation (Figure 1B). Amphiregulin protein accumulation started after 4–8 hours and peaked after 24 hours (Figure 1C). To test whether the amphiregulin release depends on new protein synthesis or on shedding of existing pro-forms, the effect of the translation inhibitors cycloheximide (=CHX) and geneticin (=G418) was investigated. Both inhibitors abrogated amphiregulin release into the supernatant, suggesting that amphiregulin release fully depends on new protein synthesis (Figure 1D). Furthermore, SCC9 cells were incubated with inhibitors for MEK and for PI3 kinase, prior to HGF stimulation. mRNA levels of amphiregulin were measured after 2 hours and protein levels were measured after 24 hours of stimulation. The inhibitor specificity and efficacy was analyzed 5 minutes after HGF stimulation and is shown in Additional file 1: Figure S1. Notably, full inhibition of amphiregulin mRNA (Figure 1E) and protein (Figure 1F) induction was achieved with the MEK inhibitor UO126, while only a minor effect was observed with the PI3K inhibitor at the protein level (Figure 1F). These experiments prove the regulation on transcript level and reveal a MAPK-pathway-dependent amphiregulin production.

**Figure 2:**
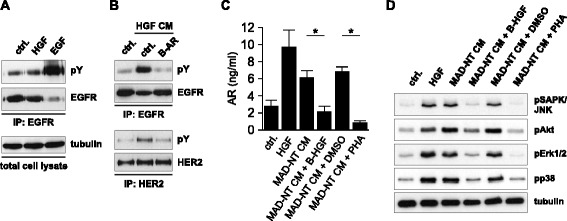
**Amphiregulin is an activator of EGFR and HER2. The CM of a monocytic cell line induces the release of amphiregulin in SCC9 cells. (A)** EGFR IP followed by Western blot analysis of SCC9 cells treated with 100 ng/ml HGF for 24 h and with 10 ng/ml EGF for 3 min. Immunoblots for phospho-tyrosine (=pY) and EGFR are shown. Total cell lysate blotted with tubulin was used as loading control. **(B)** EGFR and HER2 IP followed by Western blot analysis. SCC9 cells were stimulated for 5 min with HGF CM in the presence of 5 μg/ml amphiregulin-blocking antibody (=B-AR). The blocking antibody was added to the CM 30 min prior to stimulation. Immunoblots for pY, EGFR and HER2 are shown. **(C)** The CM of the monocytic cell line MAD-NT induced HGF-dependent amphiregulin release in SCC9 cells. Amphiregulin release was blocked, both when MAD-NT CM was pretreated with a HGF-blocking antibody (=B-HGF), as well as when SCC9 cells were pretreated for 30 min with 1 μm of the Met inhibitor PHA-665752 (=PHA). Ligand release was assayed using sandwich ELISA. Error bars indicate SEM of three independent experiments. The asterisk indicate a statistically significant decrease (*p* < 0.05, paired *t*-test). **(D)** Western blot analysis of SCC9 cells stimulated for 10 min with HGF, MAD-NT CM, MAD-NT CM plus a HGF-blocking antibody and of SCC9 cells pretreated with the Met inhibitor PHA-665752. Immunoblots for pSAPK/JNK, pAkt, pp38 MAPK and pErk1/2 are shown. Tubulin served as loading control.

Consistent with our previous findings, although the EGFR ligand amphiregulin is present in the cell culture medium, the EGF receptor does not become phosphorylated on tyrosine residues after HGF treatment [32] (Figure 2A). Therefore, in order to test if the released amphiregulin is capable of activating EGFR, the following two-step experiment was performed: first, SCC9 cells were stimulated with HGF. After 24 hours the conditioned medium (=CM) was collected. Second, fresh, untreated SCC9 cells were stimulated for three minutes with this CM, lysed and immunobloted for phospho-tyrosine and EGFR. If active EGFR ligands were shed, an activation of the EGFR should occur now. Indeed, strong EGFR and HER2 phosphorylation was observed and a blocking antibody experiment (verification of antibody specificity in Additional file 2: Figure S2) revealed amphiregulin to be the major contributor of activation (Figure 2B).

Monocytes, macrophages and fibroblasts are thought to be the major sources of HGF in the tumor stroma [35-37]. It was of interest, whether a complex CM derived from a monocytic cell line, is capable to induce EGFR ligand release. Therefore, CM of MAD-NT cells, a HGF producing subclone of the promyelocytic leukemia cell line HL60, was used (Additional file 3: Figure S3). Amphiregulin was released upon MAD-NT CM by SCC9 cells and a HGF-blocking antibody as well as the Met inhibitor PHA-665752 abrogated the amphiregulin production (Figure 2C). The Met inhibitor efficacy was analyzed 3 minutes after HGF stimulation and is shown in Additional file 4: Figure S4. In the next step, SCC9 cells were stimulated for 10 minutes with HGF and with MAD-NT CM and assayed in immunoblot analysis for the activation of the downstream signal transducers Erk1/2, Akt, p38 MAPK and SAPK/JNK. All tested signal transducers got activated by HGF and by MAD-NT CM. Both, the HGF-blocking antibody as well as the Met inhibitor PHA-665752 reduced activation of Erk1/2, Akt, p38 MAPK and SAPK/JNK (Figure 2D). These data indicate, that monocytes can be a major source of HGF and that HGF is a very potent activator of crucial signaling cascades.

**Figure 3:**
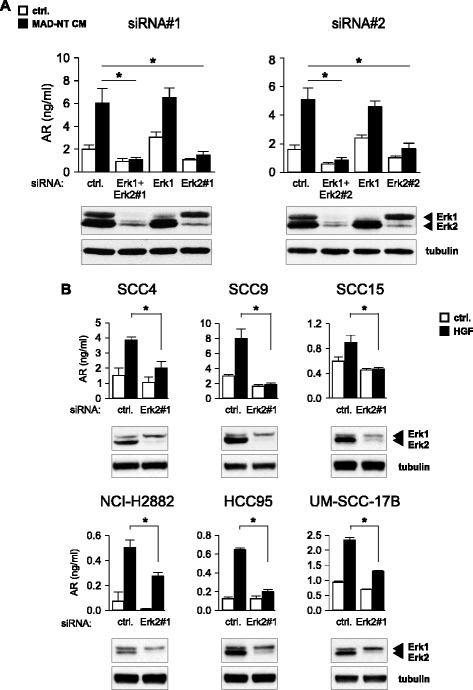
**Erk2 is required for HGF-induced amphiregulin production. (A)** Erk1 and Erk2 siRNA knockdown in SCC9 cells. MAD-NT CM was used as HGF source. For the Erk2 knockdown two siRNAs (Erk2#1 and Erk2#2) were used. Knockdown was verified with Erk1/2 immunoblot. **(B)** Erk2 siRNA knockdown in different SCC cell lines. Non-targeting siRNA was used as negative control. Amphiregulin release was measured with sandwich ELISA. Error bars indicate SEM of three independent experiments. Asterisks indicate a statistically significant decrease (*p* < 0.05, paired *t*-test). Knockdown was verified with Erk1/2 immunoblot. Tubulin served as loading control.

To evaluate which MAPK is responsible for the EGFR ligand induction, siRNA knockdown experiments of Erk1 and Erk2 were performed. The CM of MAD-NT cells was used as HGF source. A Knockdown of Erk2 dramatically reduced the release of amphiregulin, whereas a knockdown of Erk1 showed no effect. To exclude the possibility of an off-target effect, two different siRNAs for Erk2 were used. Similar results were obtained with both Erk2 siRNAs. Interestingly a double knockdown of Erk1 and Erk2 further reduced the production of amphiregulin, indicating that Erk1 could partly compensate for the loss of Erk2 (Figure 3A). Moreover Erk2 depletion blocks HGF induced amphiregulin release in tongue-, lung- and larynx-derived SCC cell lines. Notably, although in NCI-H2882 cells an Erk2 knockdown dramatically reduced basal amphiregulin production, HGF stimulation was still able to augment amphiregulin release similar to baseline levels (Figure 3B). Altogether these data suggest that in different human SCCs the upregulation and release of amphiregulin upon HGF stimulation is mediated via Erk2.

**Figure 4:**
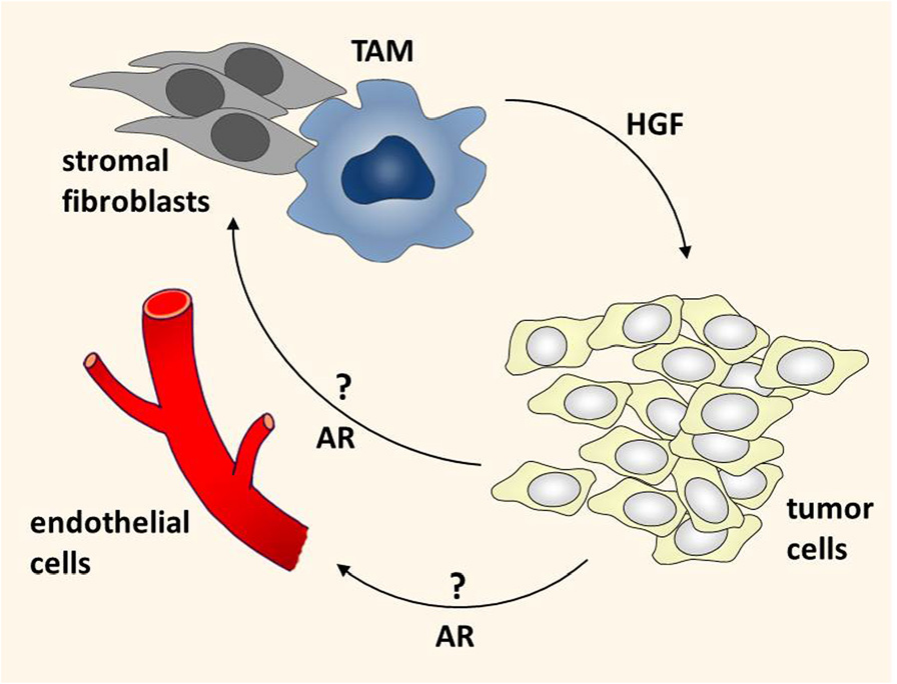
**Paracrine interaction model between TAM, tumor cells and endothelial cells.** TAMs and tumor-associated stromal fibroblasts release a variety of factors that support tumor growth and progression. HGF, one of these factors, prompts tumor cells to produce the EGFR ligand amphiregulin (AR). Importantly, once the tumor cells are activated by HGF, their EGF receptor cannot be activated by EGFR ligands anymore. However, tumor-associated endothelial cells express high levels of EGFR and have been shown to respond to EGFR activation [38,42,43]. Therefore, we propose, that the tumor vasculature represent a possible target for the produced EGFR ligands.

In this study we have shown that HGF is a strong inducer of the EGFR ligand amphiregulin in different SCC cell lines and that amphiregulin acts as a potent activator of EGFR and HER2 in HGF untreated SCC9 cells. However in HGF treated cells EGFR and HER2 cannot be further activated by amphiregulin. Therefore it is likely, that the produced amphiregulin is provided for other cell types of the tumor stroma, which did not get in contact with HGF before or amphiregulin is provided for cell types, where the EGFR is not blocked by HGF. A second possibility is that amphiregulin might induce the recruitment and proliferation of stromal cells like endothelial cells and fibroblasts (Figure 4) which promote tumor progression. Interestingly Amin et al. compared tumor-associated endothelial cells and normal endothelial cells and found that tumor-derived endothelial cells express EGFR, HER2 and HER4, whereas their normal counterparts express HER2, HER3 and HER4 [38]. As a consequence of the gain of EGFR and the loss of HER3, tumor vasculature responds to EGFR ligands. In their study they suggest that this receptor exchange promotes tumor angiogenesis. Similarly Cascone et al. showed in a mouse xenograft model of human lung andenocarcinoma an upregulation and hyperactivation of stromal EGFR in blood vessel pericytes of anti-VEGF treatment resistant tumors. In this elegant study they were able to distinguish between stromal (mouse) and cancer (human) cell specific changes of total and of phosphorylated EGFR levels [39]. Several other studies show a proangiogenic and tumor supporting effect of EGFR signaling inside the tumor vasculature [40,41]. A third possibility is, that the tumor-produced EGFR ligands exert their function in distant tissues and influence the generation of the metastatic niche. In this study, we identified MAPK signaling as the underlying pathway for new amphiregulin mRNA and protein syntheses. We intended to specifically inhibit Erk1 and 2 by siRNA knockdown and could demonstrate that Erk2 but not Erk1 is responsible for amphiregulin upregulation. In addition we demonstrate the ability of a monocytic cell line to induce amphiregulin release in a HGF/Met/MAPK-dependent manner. Our study grants further investigations which cell type in the tumor microenvironment or in distant tissues benefits from the released EGFR ligand amphiregulin during cancer progression. The detailed understanding of stromal signaling may be critical for the development of successful treatments and for the improvement of combination regimens.

## Materials and methods

### Cell culture

Human squamous cell carcinoma cell lines derived from different tissues were as follows: tongue-derived SCC4, SCC9 and SCC15, larynx-derived UM-SCC-17B and lung-derived NCI-H2882 and HCC95. The acute promyelocytic leukemia cell line HL-60 clone MAD-NT (Macrophage differentiation, non-terminal) has undergone spontaneous differentiation into semiadherent cells and was generated in our laboratory. This cell line has been used due to its ability to spontaneously produce high levels of HGF (Additional file 3: Figure S3). All cell lines were obtained from the American Type Culture Collection, except HCC95 and NCI-H2882 cells which were a kind gift from Prof. Roman Thomas (University of Cologne) and UM-SCC-17B cells which were a kind gift from Prof. Thomas Wustrow (Ludwig-Maximilian-University of Munich). The ligands and blocking antibodies were purchased from R & D Systems. Geneticin was from Invitrogen, UO126 was from Cell Signaling Technologies, cycloheximide and Wortmannin were from Sigma, PHA-665752 was from Biomol. The siRNAs for Erk1 (cat# J-003592-10), Erk2#1 (cat# J-003555-12), Erk2#2 (cat# J-003555-14) and the non-targeting siRNA (cat# D-001810-03) were purchased from Dharmacon and transfection was performed with Lipofectamine RNAiMax (Invitrogen).

### Quantitative RT-PCR

Total RNA was transcribed into complementary DNA using First Strand cDNA Synthesis Kit (Fermentas). SYBR Green Master Mix (Roche) was used for RT-qPCR measurement. The used primers were: amphiregulin → 5′-TGGTGCTGTCGCTCTTGATA-3′, ← 5′-GCCAGGTATTTGTGGTTCGT-3′; reference gene HPRT1 → 5′-GCTATAAATTCTTTGCTGACCTGCTG-3′, ← 5′-AATTACTTTTATGTCCCCTGTTGACTGG-3′.

### ELISA

Amphiregulin and HGF protein was measured using sandwich ELISA (R&D Systems).

### Immunoblotting and immunoprecipitation

For immunoprecipitation 0.5 mg of total protein, 1 μg of homemade monoclonal EGFR antibody (clone 108.1; has been characterized before [44]) and 5 μg of monoclonal HER2 antibody (clone 13D1B1; has been characterized before [44]) together with 10 μl of protein A-sepharose (GE Healthcare) were used. For the HGF immunoprecipitation 1 ml of HL60 and MAD-NT cell CM and 20 μg monoclonal HGF antibody (R&D Systems) together with 15 μl of protein A-sepharose were used. For immunoblotting the following antibodies were used: p-tyrosine (homemade clone 4G10), pAkt S473 (Cell Signaling Technologies), pErk1/2 T202/Y204 (Cell Signaling Technologies), pSAPK/JNK T183/Y185 (Cell Signaling Technologies), Erk1/2 (Santa Cruz Biotechnology), EGFR (Cell Signaling Technologies), HER2 (Millipore), HGF (R&D Systems), pMet Y1234/Y1235 (Cell Signaling Technologies), pp38 T180/Y182 (Cell Signaling Technologies) and tubulin (Sigma).

## Additional files

Additional file 1: Figure S1. Specificity and efficacy of MEK and PI3K inhibitors. Western blot analysis of SCC9 cells incubated with the MEK inhibitor UO126 and with the PI3K inhibitor wortmannin (=W) for 30 min prior to stimulation with HGF for 5 min. Total cell lysates were immunoblotted as indicated. Tubulin served as loading control. Additional file 2: Figure S2. Specificity of amphiregulin-blocking antibody. Western blot analysis of SCC9 cells treated with different EGFR ligands, which were preincubated with a amphiregulin-blocking antibody (R&D Systems) for 1 h before stimulation in a final concentration of 2.5 μg/ml. Total cell lysate was immunoblotted for pEGFR Y1173, EGFR and tubulin. EGFR and tubulin served as loading controls. Additional file 3: Figure S3. MAD-NT cells but not HL60 cells spontaneously produce high levels of HGF. (A) Quantification of HGF protein release into the supernatant of HL60 and MAD-NT cells after 24 h. Error bars indicate SEM of three independent experiments. Ligand release was assayed using sandwich ELISA. Asterisks indicate a statistically significant increase (*p* < 0.05, paired *t*-test). (B) HGF IP followed by Western blot analysis of HL60 and MAD-NT cell CM. An immunoblot for HGF is shown. Additional file 4: Figure S4. Specificity of Met inhibitor. Western blot analysis of SCC9 cells incubated with the Met inhibitor PHA-665752 for 30 min prior to stimulation with HGF for 3 min. Total cell lysates were immunoblotted for pMet. Tubulin served as loading control.

## Abbreviations

B: Blocking antibody
CHX: Cycloheximide
CM: Conditioned medium
EGF: Epidermal growth factor
EGFR: Epidermal growth factor receptor
Erk1 and Erk2: Extracellular signal-regulated kinases 1 and 2
G418: Geneticin
HER2: Human epidermal growth factor receptor 2
HGF: Hepatocyte growth factor
IP: Immunoprecipitation
MAPK: Mitogen-activated protein kinase
Met: Mesenchymal-epithelial transition factor
PI3K: Phosphoinositid-3-kinase
TAM: Tumor associated macrophage
TK: Tyrosine kinase
