# Supplementary figures and images for: Erk2 but not Erk1 regulates crosstalk between Met and EGFR in squamous cell carcinoma cell lines
Source: Mol Cancer. 2015 Mar 4;14:54. doi: 10.1186/s12943-015-0319-z (PMC4359546; doi:10.1186/s12943-015-0319-z)

## Supplementary Figure S1

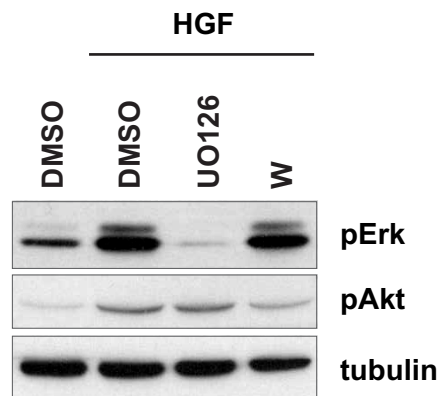

Supplement: Additional file 1: Figure S1. — Specificity and efficacy of MEK and PI3K inhibitors. Western blot analysis of SCC9 cells incubated with the MEK inhibitor UO126 and with the PI3K inhibitor wortmannin (=W) for 30 min prior to stimulation with HGF for 5 min. Total cell lysates were immunoblotted as indicated. Tubulin served as loading control. [file 12943_2015_319_MOESM1_ESM.pdf]

## Supplementary Figure S2

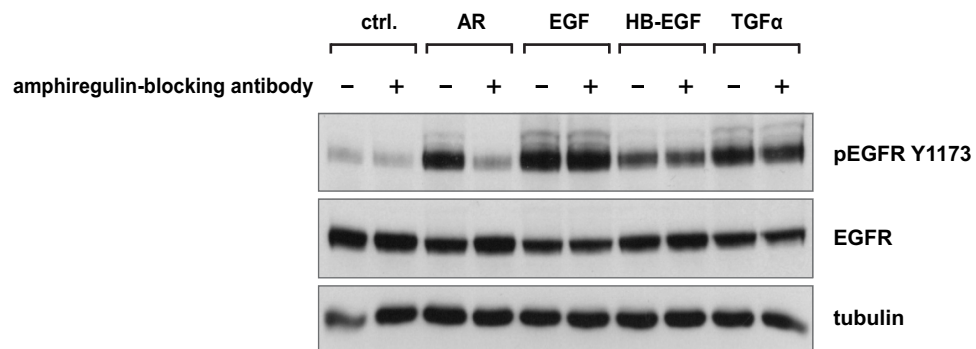

Supplement: Additional file 2: Figure S2. — Specificity of amphiregulin-blocking antibody. Western blot analysis of SCC9 cells treated with different EGFR ligands, which were preincubated with a amphiregulin-blocking antibody (R&D Systems) for 1 h before stimulation in a final concentration of 2.5 μg/ml. Total cell lysate was immunoblotted for pEGFR Y1173, EGFR and tubulin. EGFR and tubulin served as loading controls. [file 12943_2015_319_MOESM2_ESM.pdf]

## Supplementary Figure S3

**A**

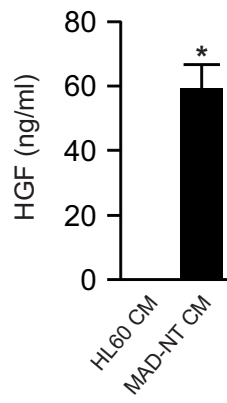

**B**

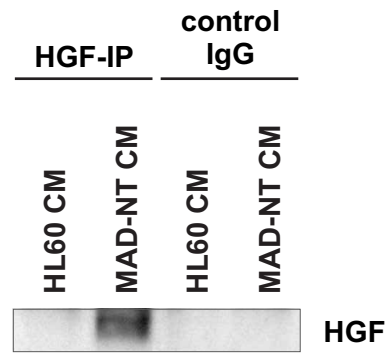

Supplement: Additional file 3: Figure S3. — MAD-NT cells but not HL60 cells spontaneously produce high levels of HGF. (A) Quantification of HGF protein release into the supernatant of HL60 and MAD-NT cells after 24 h. Error bars indicate SEM of three independent experiments. Ligand release was assayed using sandwich ELISA. Asterisks indicate a statistically significant increase (p < 0.05, paired t-test). (B) HGF IP followed by Western blot analysis of HL60 and MAD-NT cell CM. An immunoblot for HGF is shown. [file 12943_2015_319_MOESM3_ESM.pdf]

## Supplementary Figure S4

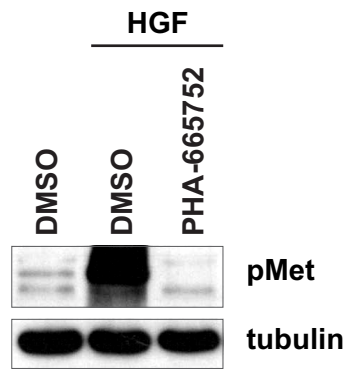

Supplement: Additional file 4: Figure S4. — Specificity of Met inhibitor. Western blot analysis of SCC9 cells incubated with the Met inhibitor PHA-665752 for 30 min prior to stimulation with HGF for 3 min. Total cell lysates were immunoblotted for pMet. Tubulin served as loading control. [file 12943_2015_319_MOESM4_ESM.pdf]
